# 

**Published:** 1788

**Authors:** 


### CONTENTS.

Page

I. *AN Account of some Experiments with Opium in the Cure of the Venereal Disease. Extracted from the Correspondence of the Military Hospitals of France, and communicated to Dr.* Simmons *by* J. F. Coste, *M. D. First Physician to the French Army,* - 7

II. *An Account of the Insect found in the Itch. From a Work lately published, in German, on the Etiology of that Disease, by* J. E. Wichmann, *M. D. Physician to His Majesty at Hanover, and Member of the Royal Society of Sciences at Goettingen, &c.* - 28

III. *Remarks on elastic Bandages. By Mr.* James Lucas, *one of the Surgeons of the General Infirmary at Leeds,* - - - 44

IV. *An Account of the Efficacy of Arsenic in Intermittents. By Mr.* J. C. Jenner, *Surgeon at Painswick, in Gloucestershire,* - - 48

V. *Account of an Amputation performed with Success in the Middle of the Foot. By Mr.* T. Turner, *jun. Surgeon at Yarmouth, in Norfolk,* - - - - - - 54

VI. *Case of an Exfoliation of the anterior Part of the Upper Jaw Bone. By Mr.* William Loftie, *Surgeon at Canterbury,* - 57

VII. *Ac-*

Page

VII. *Account of the Effects of a large Dose of Emetic Tartar; with Remarks. By* William Blackburne, *M. D. F. A. S. Member of the Royal College of Physicians, London,* - 61

VIII. *Some Account of Tipioca; and of the Species of Ipecacuanha found near Rio Janeiro,* - - - - - - 67

IX. *An Account of an Experiment lately made at Florence in a Case of Hydrophobia. Communicated by Mr.* J. Fabbroni, *Assistant Director of the Cabinet of Natural History of His Royal Highness the Grand Duke of Tuscany, and Secretary of the Royal Academy of Agriculture at Florence, in a Letter to Sir* Joseph Banks, *Bart. P. R. S. and by him to Dr.* Simmons, - - - - - 69

X. *An Experiment to determine the Effect of extirpating one Ovarium upon the Number of Young produced. By* John Hunter, *Esq. F. R. S. — From the Philosophical Transactions of the Royal Society of London,* - 71

XI. *Botanical Description of the Benjamin Tree of Sumatra. By* Jonas Dryander, *M. A. Libr. R. S. and Member of the Royal Academy of Sciences at Stockholm; communicated by Sir* Joseph Banks, *Bart. P. R. S. — From the Philosophical Transactions of the Royal Society of London,* - - - - 80

CATALOGUE OF BOOKS, - - - 83

